# Life-Space Mobility Trajectories After Elective Surgery in Older Adults

**DOI:** 10.1001/jamanetworkopen.2026.24553

**Published:** 2026-07-22

**Authors:** Nai-Wen Ku, Nelly Toledano, Nicholas Legacy, Keying Xu, Duminda N. Wijeysundera, Kristen R. Haase, Eric Pitters, Fay Bennie, Anne Stephens, Gianni R. Lorello, Kerry Kuluski, Daniel I. McIsaac, Tyler R. Chesney, Naveed Siddiqui, Karim S. Ladha, C. David Mazer, Noah Crampton, Stuart A. McCluskey, Matteo Parotto, Lisa Del Giudice, Angela Jerath, Janet van Vlymen, Su-Yin MacDonell, Melinda Davis, Alexander J. Gregory, Eric Jacobsohn, Katherine S. McGilton, Emily Hladkowicz, Shabbir M. H. Alibhai, Martine Puts

**Affiliations:** 1 Lawrence Bloomberg Faculty of Nursing, University of Toronto, Toronto, Ontario, Canada; 2 Applied Health Research Centre, Li Ka Shing Knowledge Institute, St Michael’s Hospital, Unity Health Toronto, Toronto, Ontario, Canada; 3 Department of Anesthesia, Unity Health–St Michael’s Hospital, Toronto, Ontario, Canada; 4 Applied Health Research Centre, Li Ka Shing Knowledge Institute, Unity Health Toronto, Toronto, Ontario, Canada; 5 Institute of Health Policy, Management and Evaluation, Dalla Lana School of Public Health, University of Toronto, Toronto, Ontario, Canada; 6 Department of Anesthesiology and Pain Medicine, University of Toronto, Toronto, Ontario, Canada; 7 School of Nursing, University of British Columbia, Vancouver, British Columbia, Canada; 8 Department of Anesthesiology and Pain Medicine, University of Toronto, Toronto, Ontario, Canada; 9 The Wilson Centre, Temerty Faculty of Medicine, University of Toronto, Toronto, Ontario, Canada; 10 Department of Anesthesia and Pain Management, University Health Network—Toronto Western Hospital, Toronto, Ontario, Canada; 11 Women’s College Research Institute, Toronto, Ontario, Canada; 12 Institute for Better Health, Trillium Health Partners, Mississauga, Ontario, Canada; 13 Departments of Anesthesiology and Pain Medicine, The Ottawa Hospital and University of Ottawa, Ottawa, Ontario, Canada; 14 Ottawa Hospital Research Institute, Acute Care Research Program, Ottawa, Ontario, Canada; 15 Division of General Surgery, Department of Surgery, University of Toronto, Toronto, Ontario, Canada; 16 Li Ka Shing Knowledge Institute, St Michael’s Hospital, Toronto, Ontario, Canada; 17 Department of Anesthesia and Pain Management, Sinai Health, University of Toronto, Toronto, Ontario, Canada; 18 Department of Anesthesia, Women’s College Hospital, Toronto, Ontario, Canada; 19 Department of Family Medicine, Toronto Western Hospital, University Health Network, Toronto, Ontario, Canada; 20 Department of Anesthesiology and Pain Medicine, University of Toronto, Temerty Faculty of Medicine, University of Toronto, and Department of Anesthesia and Pain Management, Toronto General Hospital, University Health Network, Toronto, Ontario, Canada; 21 Department of Family and Community Medicine, Sunnybrook Health Sciences Centre, Toronto, Ontario, Canada; 22 Department of Anesthesiology and Pain Medicine, Sunnybrook Health Sciences Centre, University of Toronto, Toronto, Ontario, Canada; 23 Department of Anesthesiology and Perioperative Medicine, Kingston Health Science Centre, Kingston, Ontario, Canada; 24 Department of Anesthesia, Providence Health Care (St Paul’s and Mount St Joseph Hospitals), Vancouver, British Columbia, Canada; 25 University of British Columbia Department of Anesthesiology, Pharmacology and Therapeutics, Vancouver, British Columbia, Canada; 26 Department of Anesthesiology, Perioperative, and Pain Medicine, University of Calgary, Calgary, Alberta, Canada; 27 Department of Anesthesiology, Perioperative and Pain Medicine and Libin Cardiovascular Institute, University of Calgary, Calgary, Alberta, Canada; 28 Department of Anesthesiology, Pain and Perioperative Medicine, University of Manitoba, Winnipeg, Manitoba, Canada; 29 KITE Research Institute: Toronto Rehabilitation Institute, University Health Network, Toronto, Ontario, Canada; 30 Department of Medicine and Department of Supportive Care, Princess Margaret Cancer Centre, University Health Network, and Faculty of Medicine and Institute for Health Policy, Management and Evaluation, University of Toronto, Toronto, Ontario, Canada

## Abstract

**Importance:**

The number of older adults living with frailty who undergo surgery is rapidly increasing worldwide. Life-space mobility (LSM) can capture physical and social dimensions of recovery and has been linked to functional decline and reduced quality of life.

**Objective:**

To examine postoperative recovery using LSM among older adults living with frailty and to identify factors associated with restricted mobility and readmission.

**Design, Setting, and Participants:**

This cohort study within the larger Functional Improvement Trajectories After Surgery study used a convergent mixed-methods exploratory design. Seventeen hospitals across Canada included older adults aged 65 years or older with a clinical frailty score (CFS) of 3 or more who underwent major elective noncardiac surgery from March 2021 to June 2023. Data were analyzed from September to October 2025.

**Exposure:**

Elective noncardiac surgery among older adults.

**Main Outcomes and Measures:**

LSM (range 0-120, with higher scores indicating more mobility) was measured at 1 to 2 months and 6 months postoperatively, with retrospective presurgery LSM captured at 1 to 2 months. Multivariable linear mixed-effects models and multivariable mixed-effects logistic regression were used to identify factors associated with LSM trajectories, restricted mobility (ie, LSM < 60), and hospital readmission.

**Results:**

This study included 204 participants (mean [SD] age, 72.8 [5.6] years; 108 males [53%]). The mean (SD) LSM was 65.1 (26.7) presurgery, 56.6 (26.7) at 2 months postsurgery, and 64.9 (25.9) at 6 months postsurgery. Eighty participants (39.2%) had restricted mobility presurgery, 114 (56.4%) at 2 months postsurgery, and 84 (41.2%) at 6 months postsurgery. Restricted mobility was associated with being a woman (odds ratio [OR], 4.72; 95% CI, 2.27-9.84), greater frailty (OR, 10.42; 95% CI, 3.56-30.49), and concurrent need for support from formal or informal caregivers (OR, 5.43; 95% CI, 2.72-10.86). Elevated-risk surgery was associated with hospital readmission at 6 months (OR, 3.18; 95% CI, 1.48-7.25).

**Conclusions and Relevance:**

In this nested cohort study, mean LSM recovered by 6 months postsurgery, but 41% of older adults still had restricted mobility. Being a woman, having greater frailty, and needing support from caregivers were associated with poor recovery. Integrating life-space assessments into individualized perioperative care planning may enhance evaluation of functional recovery, inform shared decision-making, and guide support strategies.

## Key Points

**Question:**

How does life-space mobility (LSM) change after surgery, and what factors are associated with having restricted mobility and hospital readmission at 6 months?

**Findings:**

In this cohort study of 204 participants, the mean LSM declined at 2 months and recovered by 6 months, yet 40% had restricted mobility, underscoring chronic mobility limitations, despite advances in perioperative care. Persistent homebound status was associated with women, higher frailty, and caregiver dependence.

**Meaning:**

These findings suggest that life-space assessments can better capture functional recovery after surgery and identify older patients at long-term risk of impaired mobility.

## Teaser

This cohort study examines postoperative recovery using life-space mobility among older adults living with frailty.

## Introduction

The number of older adults needing surgery is increasing worldwide due to population aging.^1^ Simultaneously, advances in surgical techniques have made it possible for more older adults to undergo surgery, including those living with frailty.^1,2^ It is estimated that approximately 40% of older adults undergoing major surgery are living with frailty.^3^ Frailty reflects vulnerability to physiological stressors like surgery and is associated with poorer functional recovery, greater risk of adverse outcomes, and higher likelihood of requiring long-term home care following surgery.^4,5,6,7,8,9^

A key consideration for older adults deciding whether to undergo invasive or major procedures is remaining independent in daily activities after discharge.^10,11,12,13,14,15^ Previous studies have shown that life-space mobility (LSM) provides a holistic measure of mobility and resilience, capturing both physical and social dimensions of recovery.^16,17^ LSM refers to the spatial area an individual moves through, how often they move, and their need for assistance with these movements.^18^ Beyond physical mobility, LSM also offers insight into the frequency with which older adults leave their homes and the places they go, thereby reflecting their social network, mental health, and participation in daily life.^19,20,21^

Poor LSM has been linked to functional decline, lower quality of life, and increased mortality in community-dwelling older adults.^18,19,20,21,22,23,24,25,26,27,28,29,30,31,32,33,34,35,36,37,38^ Understanding changes in LSM following major surgery can help assess how well older adults can continue with their daily lives after surgery. This information is crucial for both surgical decision-making and postoperative care planning. Prior research suggests that,^8,16,39^ key patient characteristics, such as gender, frailty, and cancer diagnosis, may influence mobility trajectories after surgery. For example, individuals with cancer may also follow different recovery patterns due to disease burden, psychological distress, and treatment complexity.^16,40^

Emerging evidence also indicates that unmet care needs, caregiver dependence, and surgical type play essential roles in affecting postsurgical recovery. Older adults who experience unmet needs and depend on caregivers are associated with higher health care costs and adverse outcomes.^41^ Similarly, patients undergoing high-risk surgical procedures, including intraperitoneal or suprainguinal major vascular surgeries, may face greater physiological stress, slower return to mobility, and increased health care encounters.^42^ However, LSM remains underused in surgical research, and the extent to which these factors influence recovery in older adults who are undergoing major elective surgery remains unclear. Therefore, our research questions examined how LSM changes after surgery and the factors are associated with restricted mobility and hospital readmission at 6 months?

## Methods

### Design and Participant Recruitment

This nested cohort was part of the larger Functional Improvement Trajectories (FIT) After Surgery study, which enrolled 2007 older adults (ie, aged 65 years or older) undergoing elective noncardiac surgery across 17 sites in Canada.^43^ Details of the FIT study have been published elsewhere.^43^ Our frail substudy used a convergent mixed-methods exploratory design, and in this article, we report the survey findings. The cohort study was approved by the Research Ethics Board at Unity Health Toronto (CTO Project ID: 3616) and the University of Toronto (ID 41808). All participants provided written informed consent before participation.

Participants were recruited from preoperative clinics, surgical wards, or surgeons’ offices. Eligible participants for this substudy were FIT participants with a baseline clinical frailty scale (CFS) score of 4 or more indicating frailty,^44^ discharged home or to rehabilitation, and able to provide informed consent. Due to COVID-19–related delays and the advanced stage of recruitment in the main FIT study, the threshold was broadened to CFS of 3 or more to increase the eligible pool. Participants were recruited from the FIT After Surgery Study^43^ at 1-month postsurgery, when members of the FIT team contacted them for the 1-month postsurgery follow-up. Recruitment occurred between March 16, 2021, and June 13, 2023 (eFigure 1 in Supplement 1). We used the Strengthening the Reporting of Observational Studies in Epidemiology (STROBE) reporting guideline.^45^

### Explanatory Variables

To describe the sample, basic sociodemographic data including self-reported ethnicity data using the Statistics Canada ethnicity or cultural background question,^46^ and hospital readmission data were obtained from the main FIT After Surgery study, while additional information on self-reported gender identity was collected in this substudy. Participants completed a structured survey developed by the research team (eAppendix in Supplement 1) at 1 to 2 months and 6 months postsurgery via mail or telephone, with responses recorded in REDCap. We used the definition of unmet needs from Beach et al.^41^ Unmet needs were defined within each of the basic activities of daily living (ADL) or instrumental ADL (IADL) as having difficulty, needing help, or being unable to perform the activity and being unable to perform the activity within the past month because no help was available (eg, needing help with bathing and not bathing in the absence of help).

The type of surgery risk was derived from a study by Lee et al,^42^ which developed the Revised Cardiac Risk Index (RCRI). One component of RCRI was elevated procedural risk, defined as undergoing intraperitoneal, intrathoracic, or suprainguinal major vascular surgery. The need for support from caregivers was assessed to capture the informal and formal caregiving demands during recovery. A participant was considered to have a need for support from caregivers if they received paid assistance, required a high volume of care (defined as 40 hours or more per month), were cared for by a family member, or needed help with any ADL or IADL.

### Outcome Variables

LSM was measured using the University of Alabama at Birmingham Life Space Assessment (LSA).^47^ The LSA scoring ranges from 0 (bedbound) to 120 (daily independent out-of-town mobility). It assesses mobility during the preceding 4 weeks across 5 hierarchical zones, from the bedroom (level 0) to beyond one’s hometown (level 5). The composite score is calculated by multiplying the levels reached (0 to 5) by both the frequency of the activity from 1 (ie, less than 1 time per week) to 4 (ie, daily) and the level of assistance (1 = help of another person; 1.5 = assistive device only; 2 = no assistance).

Baseline LSM was retrospectively reported at the 1- to 2-month follow-up. This measure has been previously validated using a 1- to 2-month recall window.^48^ A minimally clinically important difference (MCID) of 5 units for the LSM has been established to represent a meaningful change in functional recovery between the 2-month and 6-month intervals.^49^ We used the published score of LSM less than 60 to indicate restricted mobility.^34,47,50,51^ Hospital readmission within 6 months was also examined.

### Statistical Analysis

Data were analyzed using R version 4.4.2 (R Project for Statistical Computing). Descriptive statistics summarized sample characteristics, LSM scores, and caregiving measures. Based on prior literature, a sample size of 329 was estimated to detect a MCID of 5.0 points in LSM between 2 and 6 months, assuming a conservative SD of 31.5, power of 80%, and 2-sided α of .05.^16,49^ However, due to delays caused by COVID-19, by the time the frail substudy started, recruitment for the main FIT study was over halfway complete. Ultimately, 204 participants completed the 2-month survey, and 198 completed the 6-month follow-up (eMethods in Supplement 1).

To assess change in LSM over time, we examined 2 intervals: baseline (assessed retrospectively) to 1 to 2 months postsurgery and 2 to 6 months postsurgery. Paired *t* tests were used for unadjusted comparisons. Linear mixed-effects models with a random intercept to account for clustering over time with patients were conducted. Baseline LSM response was included in the outcome vector, an approach appropriate for observational designs.^52^ Parameter estimates, 95% CIs, and *P* values were reported. Interaction terms between gender and other covariates (time, frailty, caregiver support, and elevated-risk surgery) were included to assess whether changes in LSM or associations with restricted mobility differed across these subgroups.^16,39^

To identify factors associated with restricted mobility (LSM <60) at 6 months, we used multivariable mixed-effects logistic regression with a random intercept to account for within-patient clustering over time. Odds ratios (ORs), 95% CIs, and *P* values were reported. Finally, to explore the association between baseline LSM and hospital readmission within 6 months, we conducted exploratory logistic regression analyses with baseline LSM and the number of unmet needs as independent variables. All models adhered to a standard, theory-based approach. Multicollinearity was examined using variance inflation factor and ranged from 1.03 to 2.07, indicating no issues. Sensitivity analyses were conducted adding level of education and living alone or not to the 3 models. The data analysis took from September to October 2025.

## Results

This study included 204 participants (mean [SD] age, 72.8 [5.6] years; 108 males [52.9%] (Table 1 and eFigure 1 in Supplement 1). Ninety participants (44.1%) underwent cancer surgery (Table 1). Participants’ reported gender identity was consistent with their sex assigned at birth. Based on the CFS, 85 participants (41.7%) were classified as managing well (ie, CFS 3), 83 (40.7%) as living with very mild frailty (ie, CFS 4), and 36 (17.6%) as living with mild to moderate frailty (ie, CFS ≥5). Forty-one participants (20.2%) had unmet needs 1- to 2-months postsurgery and 21 (10.6%) at 6 months after surgery. At 1- to 2-months postsurgery, 151 (74.4%) reported the need for support from formal and informal caregivers and 105 (53.0%) at 6 months postsurgery.

**Table 1.** Baseline Demographics and Clinical Characteristics of Older Adults

Characteristic	Participants, No. (%)				
	Total sample	Men	Women	2-mo Restricted mobility	6-mo Restricted mobility
Total, No. (%)	204 (100)	108 (52.9)	96 (47.1)	115 (56.4)	84 (41.2)
Age, mean (SD), y	72.8 (5.6)	72.9 (5.3)	72.8 (5.9)	73.1 (5.6)	73.7 (5.9)
Education					
At least some university or college	155 (76.0)	78 (72.2)	77 (80.2)	81 (71.1)	58 (69.1)
Did not complete high school	17 (8.3)	10 (9.3)	7 (7.3)	13 (11.4)	8 (9.5)
Completed high school	31 (15.2)	20 (18.5)	11 (11.5)	20 (17.5)	17 (20.2)
Prefer not to say	1 (0.5)	0	1 (1.0)	1 (0.9)	1 (1.2)
Ethnicity					
White	189 (92.6)	102 (94.4)	87 (90.6)	106 (92.2)	74 (88.1)
Other^a^	14 (6.9)	6 (5.6)	8 (8.3)	9 (7.8)	10 (11.9)
Prefer not to say	1 (0.5)	0	1 (1.0)	0	0 (
Living at home alone	53 (26.0)	19 (17.6)	34 (35.4)	37 (32.5)	24 (28.6)
Cognitive impairment presurgery	27 (13.8)	14 (13.9)	13 (13.8)	19 (17.1)	15 (18.8)
Clinical frailty scale median					
Median (IQR)	4.0 (3.0-4.0)	4.0 (3.0-4.0)	4.0 (3.0-4.0)	4.0 (3.0-4.5)	4.0 (3.0-5.0)
3	85 (41.7)	49 (45.4)	36 (37.5)	45 (39.1)	25 (29.8)
4	83 (40.7)	43 (39.8)	40 (41.7)	41 (35.7)	33 (39.3)
≥5	36 (17.6)	16 (14.8)	20 (20.8)	29 (25.2)	26 (31.0)
Malignancy					
No	98 (48.0)	53 (49.1)	45 (46.9)	55 (47.8)	42 (50.0)
Yes, unrelated to surgery	16 (7.8)	8 (7.4)	8 (8.3)	10 (8.7)	8 (9.5)
Yes, indication for surgery	90 (44.1)	47 (43.5)	43 (44.8)	50 (43.5)	34 (40.5)
Elevated risk surgery	101 (49.5)	46 (42.6)	55 (57.3)	58 (50.4)	40 (47.6)
No. of multimorbid conditions					
0	14 (6.9)	8 (7.4)	6 (6.2)	6 (5.3)	5 (6.0)
1	35 (17.2)	16 (14.8)	19 (19.8)	22 (19.3)	12 (14.3)
2	59 (28.9)	30 (27.8)	29 (30.2)	35 (30.7)	23 (27.4)
3	38 (18.6)	19 (17.6)	19 (19.8)	19 (16.7)	16 (19.0)
≥4	57 (28.14)	34 (31.8)	23 (24.0)	32 (28.1)	28 (33.3)
Surgery type					
Vascular procedures	11 (5.4)	9 (8.3)	2 (2.1)	5 (4.3)	3 (3.6)
Intrathoracic procedures	15 (7.4)	8 (7.4)	7 (7.3)	9 (7.8)	5 (6.0)
Intraperitoneal procedures	85 (41.7)	38 (35.2)	47 (49.0)	48 (41.7)	34 (40.5)
Orthopedic, spine procedures	47 (23.0)	22 (20.4)	25 (26.0)	29 (25.2)	25 (29.8)
Urologic or gynecological procedures	36 (17.6)	25 (23.1)	11 (11.5)	18 (15.7)	15 (17.9)
Head-and-neck procedures	8 (3.9)	5 (4.6)	3 (3.1)	5 (4.3)	2 (2.4)
Other procedure types	2 (1.0)	1 (0.9)	1 (1.0)	1 (0.9)	0
Unmet needs^b^					
1 to 2 mo	41 (20.2)	20 (18.7)	21 (21.9)	30 (26.1)	24 (28.6)
6 mo	21 (10.6)	8 (7.7)	13 (13.8)	16 (14.5)	14 (16.7)
Need for support from caregivers					
1 to 2 mo	151 (74.4)	77 (72.0)	74 (77.1)	102 (88.7)	70 (83.3)
6 mo	105 (53.0)	46 (44.2)	59 (62.8)	78 (70.9)	63 (75.0)
LSM, mean (SD)					
Presurgery	65.1 (26.7)	70.5 (26.8)	59.0 (25.4)	51.8 (23.6)	50.64 (23.5)
1- to 2-mo Postsurgery	56.6 (26.7)	63.1 (27.3)	49.3 (24.1)	36.9 (13.9)	43.3 (21.9)
6-mo Postsurgery	64.9 (25.9)	71.5 (24.3)	57.5 (25.7)	53.4 (23.7)	40.8 (15.1)
Restricted mobility (LSM <60)					
Presurgery	80 (39.2)	29 (26.9)	51 (53.1)	NA	NA
1- to 2-mo Postsurgery	114 (56.4)	45 (42.5)	69 (71.9)	NA	NA
6-mo Postsurgery	84 (41.2)	36 (35.0)	48 (51.1)	NA	NA
Hospital readmission within 6 mo	39 (21.2)	20 (20.2)	19 (22.4)	20 (18.9)	15 (19.7)

Abbreviations: LSM, life-space mobility; NA, not applicable.

^a^ Black, Chinese, Indigenous, Latin American, Norwegian, South Asian, Southeast Asian, Ukrainian, and unknown.

^b^ Unmet needs were defined within each of the basic activities of daily living or instrumental activities of daily living as having difficulty, needing help, or being unable to perform the activity and, within the past month, being unable to perform the activity because no help was available.

Figure, A shows changes in LSM status over time based on the MCID. At presurgery, 124 participants (60.8%) had unrestricted mobility (ie, LSM ≥60). By 6 months, 99 participants (48.5%) were classified as improved, 52 (25.5%) as unchanged, and 47 (23.0%) as declined. Figure, B depicts changes in restricted mobility by gender over time, with a high proportion of women at restricted mobility across time points. eFigure 2 in Supplement 1 illustrates restricted mobility by the CFS group over time. Overall, 70 participants (34.5%) of participants had both frailty (ie, CFS ≥4) and restricted mobility at 2 months postsurgery and 59 participants (29.8%) at 6 months postsurgery.

**Figure.**
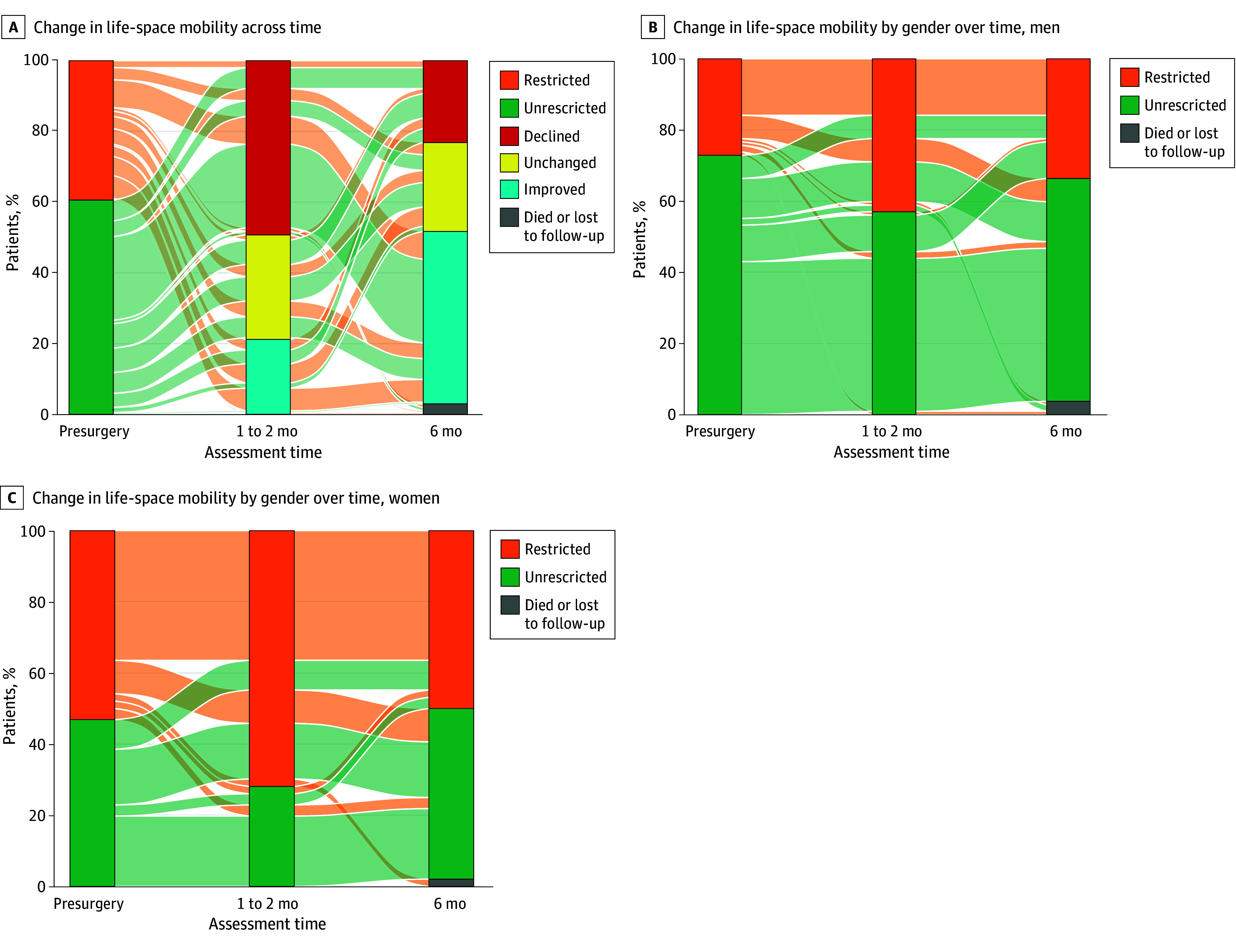
Change in Life-Space Mobility From Presurgery to 2 Months and 6 Months After Surgery Improved or declined indicates the change value exceeded the minimally clinically important difference threshold of ±5 points; unchanged, a change value smaller than the minimally clinically important difference threshold of ±5 points; restricted, life-space mobility less than 60; unrestricted, life-space mobility 60 or greater.

eFigure 3 in Supplement 1 displays mean changes in LSM trajectories by frailty level, gender, and cancer diagnosis. Overall, mean (SD) LSM was 65.2 (26.7) presurgery, 56.6 (26.7) at 2 months postsurgery and 64.9 (25.9) 6 months postsurgery. Participants with frailty (CFS ≥5) had the lowest LSM scores across time points. Gender differences were apparent: women consistently had lower LSM scores than men. Among women, the mean (SD) score was 59.0 (25.4) presurgery, 49.3 (24.1) at 2 months postsurgery, and 57.5 (25.7) at 6 months postsurgery. LSM trajectories were similar for participants with and without cancer, with comparable scores at baseline (mean [SD], 67.4 [25.9] vs 62.5 [27.4]) and at 6 months postsurgery (65.6 [26.0] vs 64.3 [25.8]).

Table 2 summarizes the results of the linear mixed-effects model. Gender, higher frailty status, and the need for support from caregivers were significantly associated with lower LSM scores. Women had LSM scores 11.83 (95% CI, –17.4 to –6.3) points lower than men. Participants classified as living with moderate to severe frailty (CFS ≥5) scored 15.7 (95% CI, –23.5 to –7.8) points lower than those with a CFS of 3. These findings indicate that higher frailty and identifying as a woman were associated with poorer mobility recovery. Participants after surgery requiring formal or informal caregiver support had LSM scores 12.2 (95% CI, –16.4 to –8.0) points lower on average compared with those who did not. In contrast, at 6-month postsurgery, a significant improvement in LSM was observed (mean increase, 6.1 [95% CI, 2.2 to 9.9] points), compared with baseline, after controlling for all other covariates.

**Table 2.** Linear Mixed-Effects Model for Life-Space Mobility Within 6 Months

Characteristic	Estimated coefficient (95% CI)	*P* value
Gender^a^		
Men	1 [Reference]	NA
Women	−11.83 (−17.38 to −6.27)	<.001
Age (per 5-y increase)	−1.75 (−4.22 to 0.72)	.17
CFS^b^		
3	1 [Reference]	NA
4	−1.49 (−7.54 to 4.55)	.63
≥5	−15.66 (−23.51 to −7.80)	<.001
Elevated risk surgery^c^		
No	1 [Reference]	NA
Yes	3.99 (−1.61 to 9.59)	.16
Time		
Presurgery	1 [Reference]	NA
1 to 2 mo	0.58 (−3.85 to 5.01)	.80
6 mo	6.05 (2.17 to 9.93)	.002
Need for support from formal or informal caregivers^d^		
No	1 [Reference]	NA
Yes	−12.23 (−16.44 to −8.01)	<.001

Abbreviations: CFS, clinical frailty score; NA, not applicable.

^a^ The likelihood ratio test indicates no evidence (likelihood ratio test *P* value = 0.834) for the interaction effect between gender and time on life-space mobility.

^b^ The likelihood ratio test indicates statistically significant evidence (likelihood ratio test *P* value = 0.043) for the interaction effect between gender and elevated risk surgery on life-space mobility.

^c^ The likelihood ratio test indicates no evidence (likelihood ratio test *P* value = 0.603) for the interaction effect between gender and Clinical Frailty Scale on life-space mobility.

^d^ The likelihood ratio test indicates no evidence (likelihood ratio test *P* value = 0.235) for the interaction effect between gender and the need for support from caregivers on life-space mobility.

Table 3 presents the results from the multivariable mixed-effects logistic regression model for restricted mobility (LSM <60) at 6 months. Being a woman was associated with a 4.7 (95% CI, 2.3 to 9.8)–fold increase in odds of restricted mobility compared with men. Participants classified as living with moderate to severe frailty (CFS ≥5) had 10.4 (95% CI, 3.6 to 30.5) times the odds of restricted mobility compared with those with a CFS of 3. Participants with the need for support from formal or informal caregivers had 5.43 (95% CI, 2.7 to 10.9) times the odds of restricted mobility. The likelihood of restricted mobility decreased at 6 months (OR, 0.5 [95% CI, 0.3 to 1.0]), indicating some functional recovery over time.

**Table 3.** Multivariable Mixed-Effects Logistic Regression for Restricted Mobility (Life-Space Mobility <60) Within 6 Months

Level	Odds ratio (95% CI)	*P* value
Gender		
Men	1 [Reference]	NA
Women	4.73 (2.27-9.84)	<.001
Age (per 5-y increase)	1.29 (0.94-1.76)	.11
CFS		
3	1 [Reference]	NA
4	1.46 (0.70-3.08)	.32
≥5	10.42 (3.56-30.49)	<.001
Elevated risk surgery		
No	1 [Reference]	NA
Yes	0.68 (0.34-1.36)	.28
Time		
Presurgery	1 [Reference]	NA
1 to 2 mo	0.98 (0.46-2.09)	.97
6 mo	0.49 (0.25-0.97)	.04
Need for support from formal or informal caregivers		
No	1 [Reference]	NA
Yes	5.43 (2.72-10.86)	<.001

Abbreviations: CFS, clinical frailty score; NA, not applicable.

Table 4 shows variables associated with hospital readmission within 6 months. Participants who underwent elevated-risk surgery had 3.2 (95% CI, 1.5 to 7.3) times the odds of readmission. Baseline LSM was not associated with higher odds of readmission (OR, 1.01 [95% CI, 0.99-1.03), which may have limited statistical power due to small number of readmission events (n = 39), resulting in a reduced ability to detect modest associations. The results of the sensitivity analyses including education and living at home alone as covariates were very similar (eTables 1 to 3 in Supplement 1).

**Table 4.** Multivariable Logistic Regression Model for Hospital Readmission Within 6 Months (n = 39)

Level	Odds ratio (95% CI)	*P* value
Unmet needs		
No	1 [Reference]	NA
Yes	2.05 (0.85-4.87)	.11
Presurgery life-space mobility	1.01 (0.99-1.03)	.24
CFS		
3	1 [Reference]	NA
4	0.78 (0.34-1.78)	.55
≥5	0.88 (0.26-2.74)	.83
Elevated risk surgery		
No	1 [Reference]	1 [Reference]
Yes	3.09 (1.43-7.06)	.004

Abbreviations: CFS, clinical frailty score; NA, not applicable.

## Discussion

In this nested cohort study, the mean LSM recovered by 6 months postsurgery, a substantial proportion of older adults had restricted mobility. Being a woman, having higher frailty level, and needing support from formal or informal caregivers were independently associated with restricted mobility; and although baseline LSM was not significantly associated with hospital readmission, its clinical relevance should not be overlooked. These findings highlight persistent limitations in functional recovery, despite advances in perioperative care.

Despite an average recovery to nearly baseline, 40% still experienced restricted mobility in 40% at 6 months. This finding aligns with Stewart et al,^16^ who reported a temporary decline in LSM at 6 weeks following urogynecological or gynecologic-oncologic surgery, with partial recovery by 6 months. Our study extends this knowledge by demonstrating similar patterns in a larger population with different degrees of frailty undergoing a broader range of elective surgeries. Consistent with Stewart et al,^16^ we also observed no meaningful difference in mobility recovery between participants with and without a cancer diagnosis. Notably, a substantial proportion of participants who were women, compared with men, did not return to their baseline by 6 months. Moreover, although older adults with higher frailty levels showed some improvement from baseline, many continued to experience restricted mobility. These findings emphasize the need for clinicians to focus more on older patients living with frailty and other risk factors, rather than solely on those with cancer diagnosis.

We identified 3 patient-level factors independently associated with restricted mobility at 6 months: being a woman, living with greater frailty, and the need for support from formal or informal caregivers. Women consistently had lower LSM scores across all time points and had nearly 5 times the odds of restricted mobility compared with men, even after adjusting for other variables. This finding is consistent with prior studies showing that older women are more likely to experience mobility limitations.^39,53^ Traditional gender norms may limit outings, travel distances, and participation in activities, while increasing time spent on housework and heightening caregiving burdens, thereby restricting postoperative mobility.^39,53^ Differences in marital status, social network size, and socioeconomic resources may also contribute to gender disparities in mobility.^54^ As previously reported, frailty was also strongly associated with lower LSM scores and persistent mobility restrictions.^7,55^ These findings underscore the importance of frailty screening as a crucial component of perioperative risk stratification. The need for formal or informal caregiver support was linked to lower mobility, likely reflecting broader social and functional dependence.^56^ These findings underscore the critical role of caregiving demands in shaping recovery trajectories and reinforcing the importance of transitional, postdischarge, and community care support, including continuity of care, coordinated discharge planning, and rehabilitation resources that address the needs of both older adults and their caregivers.^57^

Although the baseline LSM was not statistically significantly associated with 6-month readmission, likely due to limited power from the small number of events, it remains an important measure, as it provides a holistic, patient-centered assessment of functional recovery and social engagement that goes beyond traditional clinical outcomes.^17,18^ Undergoing elevated-risk surgery was significantly associated with increased odds of hospital readmission within 6 months. This finding was aligned with existing literature^42^ and has also been linked to days not spent at home.^58,59,60^ Individuals undergoing these procedures have greater care complexity, require more intensive follow-up, and may have experienced disruption in the usual care pathway due to the COVID-19 pandemic, all of which could contribute to hospital readmission.^61^

Several clinical implications should be considered. Incorporating life-space assessments into surgical care may be warranted, as LSM captures overall functional change that may be overlooked by conventional functional status measures (eg, ADL or IADLs) during the perioperative period.^16^ Routine assessment of frailty and identification of high-risk surgical types may help clinicians recognize older individuals at risk of prolonged recovery and adverse outcomes, thereby supporting the use of comprehensive geriatric assessments and tailored management strategies. Future interventions should integrate considerations of gendered roles, frailty, and formal or informal caregiver dependence into physical rehabilitation programs, community-based supports, and discharge planning, as these factors play a key role in improving life-space mobility in later life.

### Limitations

This study has some limitations. Due to COVID-19–related delays, we revised our frailty threshold to include participants with CFS of 3 or more and were only able to recruit 62% of our target sample. The distribution of CFS scores did not differ significantly between participants and nonparticipants (*P* = .70). This smaller sample size likely contributed to a wider 95% CI. In addition, our smaller sample size may have led to reduced statistical power to detect significant associations, potentially resulting in Type II errors. Although the response rate among eligible individuals who agreed to be contacted was only 42%, there were no significant differences in CFS scores between participants and nonparticipants. However, selection bias remains possible. Some individuals may have declined participation because they felt too unwell or overwhelmed after surgery. Participants were limited to English speakers, so our findings may not be generalizable to the broader older surgical population.

## Conclusions

In this study, although LSM improved by 6 months, many older adults with frailty still experienced restricted mobility. In

tegrating LSM into surgical care may better measure functional recovery. Women, those who need for support from caregivers, and individuals with higher frailty are key risk factors for poor mobility recovery. Future research is warranted to prioritize mobility restoration and incorporate tailored strategies to support these high-risk groups.

## Notes

Supplement 1. **eAppendix.** Survey questionnaire
eMethods.
**eFigure 1.** Flow chart overview of recruitment and retention of older adults (n=205) **eFigure 2.** Life-space mobility (LSM) status by clinical frailty scale (CFS) group from presurgery to 2 and 6 months after surgery **eFigure 3. **Mean changes in life space mobility (LSM) over time and stratified by frailty, gender, and cancer diagnosis **eTable 1.** Linear mixed-effect model for life-space mobility (LSM) within 6 months now including education and living at home alone vs not (sensitivity analysis) **eTable 2.** Multivariable mixed-effects logistic regression for restricted mobility (LSM < 60) within 6 months now including education and living at home alone vs not (sensitivity analyses) **eTable 3.** Multivariable logistic regression model for hospital readmission within 6 months (n=38) now including education and living at home alone vs not (sensitivity analyses)

Supplement 2.
Data Sharing Statement
