# Supplementary material for: Life-Space Mobility Trajectories After Elective Surgery in Older Adults
Source: JAMA Netw Open. 2026 Jul 22;9(7):e2624553. doi: 10.1001/jamanetworkopen.2026.24553 (PMC13392812; doi:10.1001/jamanetworkopen.2026.24553)
Supplement: Supplement 1. — eAppendix. Survey questionnaire eMethods. eFigure 1. Flow chart overview of recruitment and retention of older adults (n=205) eFigure 2. Life-space mobility (LSM) status by clinical frailty scale (CFS) group from presurgery to 2 and 6 months after surgery eFigure 3. Mean changes in life space mobility (LSM) over time and stratified by frailty, gender, and cancer diagnosis eTable 1. Linear mixed-effect model for life-space mobility (LSM) within 6 months now including education and living at home alone vs not (sensitivity analysis) eTable 2. Multivariable mixed-effects logistic regression for restricted mobility (LSM < 60) within 6 months now including education and living at home alone vs not (sensitivity analyses) eTable 3. Multivariable logistic regression model for hospital readmission within 6 months (n=38) now including education and living at home alone vs not (sensitivity analyses) [file jamanetwopen-e2624553-s001.pdf]

Supplemental Online Content

Ku NW, Toledano N, Legacy N, et al. Life space mobility trajectories after elective surgery in older adults. *JAMA Netw Open*. 2026;9(7):e2624553.
doi:10.1001/jamanetworkopen.2026.24553

eAppendix. Survey Questionnaire

eMethods.

eFigure 1. Flow chart overview of recruitment and retention of older adults (n=205)

eFigure 2. Life-space mobility (LSM) status by clinical frailty scale (CFS) group from presurgery to 2 and 6 months after surgery

eFigure 3. Mean changes in life space mobility (LSM) over time and stratified by frailty, gender, and cancer diagnosis

eTable 1. Linear mixed-effect model for life-space mobility (LSM) within 6 months now including education and living at home alone vs not (sensitivity analysis)

eTable 2. Multivariable mixed-effects logistic regression for restricted mobility (LSM < 60) within 6 months now including education and living at home alone vs not (sensitivity analyses)

eTable 3. Multivariable logistic regression model for hospital readmission within 6 months (n=38) now including education and living at home alone vs not (sensitivity analyses)

This supplemental material has been provided by the authors to give readers additional information about their work.

© 2026 Ku NW et al. *JAMA Network Open*.

eAppendix. Survey questionnaire (The 6-month survey questionnaire is similar)

Survey for older adult at 2 months after surgery

1. What is your current gender identity ?

- ☐ Male
- ☐ Female
- ☐ Trans male/Trans man
- ☐ Trans female/Trans woman
- ☐ Genderqueer/ Gender non-conforming
- ☐ Different identity: ____________________(please specify)
- ☐ Prefer not to say

2. What type of dwelling do you currently live in?

- ☐ House (single detached, semi-detached, duplex or townhouse)
- ☐ Apartment or condominium
- ☐ Seniors' housing (retirement home, assisted living)
- ☐ Institution (old age facility)
- ☐ Hotel, rooming or lodging house

3. Does your home/apartment/unit/room have a:

a) Ramp yes ☐ /no ☐
b) Elevator yes ☐ /no ☐
c) Stair life or stair glide yes ☐ /no ☐
d) Grab bar in the shower or tub area yes ☐ /no ☐
e) Seat for in the shower or tub yes ☐ /no ☐
f) Raised toilet or raised toilet seat? yes ☐ /no ☐
g) Grab bars around the toilet yes ☐ /no ☐

4. There are a few devices that help with daily activities.
In the last month did you use:

a) Glasses or other vision aids such as a magnifying glass? yes ☐ /no ☐
b) A hearing aid? yes ☐ /no ☐
c) A cane? yes ☐ /no ☐
d) A walker? yes ☐ /no ☐
e) A wheelchair? yes ☐ /no ☐
f) A scooter? yes ☐ /no ☐
g) A reacher or grabber to pick up things more easily? yes ☐ /no ☐
h) Special items to help with dressing such as button hooks or clothes that are designed to get on and off easily?
Adapted utensils to help you to eat or cut your food (like easy-to-grip silverware, knives that rock etc.). yes ☐ /no ☐

© 2026 Ku NW et al. *JAMA Network Open*.

5 **Can you use the telephone ...**

- ☐ without help, including looking up numbers and dialing;
- ☐ with some help (can answer phone or dial operator in an emergency, but need a special phone or help in getting the number or dialing); or
- ☐ are you completely unable to use the telephone?

6. **Can you get to places out of walking distance ...**

- ☐ without help (drive your own car, or travel alone on buses, or taxis);
- ☐ with some help (need someone to help you or go with you when traveling); or
- ☐ are you unable to travel unless emergency arrangements are made for a specialized vehicle like an ambulance?

7. **Can you go shopping for groceries or clothes [ASSUMING YOU HAVE TRANSPORTATION]** ...

- ☐ without help (taking care of all shopping needs yourself, assuming you had transportation);
- ☐ with some help (need someone to go with you on all shopping trips); or
- ☐ are you completely unable to do any shopping?
- ☐ Not applicable (never do, not related to health)

8. **Can you prepare your own meals ...**

- ☐ without help (plan and cook full meals yourself);
- ☐ with some help (can prepare some things but unable to cook full meals yourself);
- ☐ are you completely unable to prepare any meals?
- ☐ Not applicable (never do, not related to health)

9. **Can you do your housework ...**

- ☐ without help (can clean floors, etc.);
- ☐ with some help (can do light housework but need help with heavy work); or
- ☐ are you completely unable to do any housework?
- ☐ Not applicable (never do, not related to health)

© 2026 Ku NW et al. *JAMA Network Open*.

10. **Can you take your own medicine ...**

- ☐ without help (in the right doses at the right time);
- ☐ with some help (able to take medicine if someone prepares it for you and/or reminds you to take it); or
- ☐ are you completely unable to take your medicines?

11. **Can you handle your own money ...**

- ☐ without help (write checks, pay bills, etc.);
- ☐ with some help (manage day-to-day buying but need help with managing your check book and paying your bills); or
- ☐ are you completely unable to handle money?
- ☐ Not applicable (never do, not related to health)

12. **Can you eat ...**

- ☐ without help (able to feed yourself completely);
- ☐ with some help (need help with cutting, etc.); or
- ☐ are you completely unable to feed yourself?

13. **Can you dress and undress yourself ...**

- ☐ without help (able to pick out clothes, dress and undress yourself);
- ☐ with some help; or
- ☐ are you completely unable to dress and undress yourself?

14. **Can you take care of your own appearance, for example combing your hair**

- ☐ without help;
- ☐ with some help; or
- ☐ are you completely unable to maintain your appearance yourself?

15. **Can you walk ...**

- ☐ without help (except from a cane);
- ☐ with some help from a person or with the use of a walker, or crutches,
  etc.; or
- ☐ are you completely unable to walk?

16. **Can you get in and out of bed ...**

- ☐ without any help or aids;
- ☐ with some help (either from a person or with the aid of some device); or
- ☐ are you totally dependent on someone else to lift you?

© 2026 Ku NW et al. *JAMA Network Open*.

17. **Can you take a bath or shower ...**

- ☐ without help;
- ☐ with some help (need help getting in and out of the tub, or need special attachments on the tub); or
- ☐ are you completely unable to bathe yourself?

18. During the past four weeks, have you been to other rooms of your home besides the room where you sleep?

- ☐ Yes
- ☐ No (please go to question 33)
- ☐ Don't know

19. How often did you get to other rooms of your home besides the room where you sleep?

- ☐ Less than once a week
- ☐ 1-3 times per week
- ☐ 4-6 times per week
- ☐ Daily
- ☐ Don't know

20. During the past four weeks, have you been to an area outside your home such as your porch, deck or patio, hallway (of an apartment building) or garage, in your own yard or driveway?

- ☐ Yes
- ☐ No (please go to question 22)
- ☐ Don't know

21. How often did you get to an area outside your home such as your porch, deck or patio, hallway (of an apartment building) or garage, in your own yard or driveway?

- ☐ Less than once a week
- ☐ 1-3 times per week
- ☐ 4-6 times per week
- ☐ Daily
- ☐ Don't know

© 2026 Ku NW et al. *JAMA Network Open*.

22. During the past four weeks, have you been to places in your neighbourhood, other than your own yard or apartment building?

- ☐ Yes
- ☐ No (please go to question 24)
- ☐ Don't know

23. How often did you get to places in your neighbourhood, other than your own yard or apartment building?

- ☐ Less than once a week
- ☐ 1-3 times per week
- ☐ 4-6 times per week
- ☐ Daily
- ☐ Don't know

24. During the past four weeks, have you been to places outside your neighbourhood, but within your town?

- ☐ Yes
- ☐ No (please go to question 26)
- ☐ Don't know

25. How often did you get to places outside your neighbourhood, but within your town?

- ☐ Less than once a week
- ☐ 1-3 times per week
- ☐ 4-6 times per week
- ☐ Daily
- ☐ Don't know

26. During the past four weeks, have you been to places outside your town?

- ☐ Yes
- ☐ No (please go to question 28)
- ☐ Don't know

27. How often did you get to places outside your town?

- ☐ Less than once a week
- ☐ 1-3 times per week
- ☐ 4-6 times per week
- ☐ Daily
- ☐ Don't know

© 2026 Ku NW et al. *JAMA Network Open*.

28. Did you use aids or equipment, or need help from another person to get to other rooms of your home besides the room where you sleep?

- ☐ Yes personal assistance
- ☐ Yes equipment only
- ☐ No
- ☐ Don't know

29. Did you use aids or equipment, or need help from another person to get to an area outside your home such as your porch, deck or patio, hallway (of an apartment building) or garage, in your own yard or driveway?

- ☐ Yes personal assistance
- ☐ Yes equipment only
- ☐ No
- ☐ Did not leave my home
- ☐ Don't know

30. Did you use aids or equipment, or need help from another person to get to places in your neighbourhood, other than your own yard or apartment building?

- ☐ Yes personal assistance
- ☐ Yes equipment only
- ☐ No
- ☐ Did not leave my home
- ☐ Don't know

31. Did you use aids or equipment, or need help from another person to get to places outside your neighbourhood, but within your town?

- ☐ Yes personal assistance
- ☐ Yes equipment only
- ☐ No
- ☐ Did not leave my neighbourhood
- ☐ Don't know

32. Did you use aids or equipment, or need help from another person to get to places outside your town?

- ☐ Yes personal assistance
- ☐ Yes equipment only
- ☐ No
- ☐ Did not leave my town
- ☐ Don't know

© 2026 Ku NW et al. *JAMA Network Open*.

We would like to ask the same questions again, _*but thinking back about the 4 weeks prior to your surgery*_

33. During the four weeks prior to your surgery, had you been to other rooms of your home besides the room where you sleep?

- ☐ Yes
- ☐ No (please go to question 48)
- ☐ Don't know

34. How often did you get to other rooms of your home besides the room where you sleep?

- ☐ Less than once a week
- ☐ 1-3 times per week
- ☐ 4-6 times per week
- ☐ Daily
- ☐ Don't know

35. During the four weeks prior to your surgery, had you been to an area outside your home such as your porch, deck or patio, hallway (of an apartment building) or garage, in your own yard or driveway?

- ☐ Yes
- ☐ No (please go to question 37)
- ☐ Don't know

36. How often did you get to an area outside your home such as your porch, deck or patio, hallway (of an apartment building) or garage, in your own yard or driveway?

- ☐ Less than once a week
- ☐ 1-3 times per week
- ☐ 4-6 times per week
- ☐ Daily
- ☐ Don't know

37. During the four weeks prior to your surgery, had you been to places in your neighbourhood, other than your own yard or apartment building?

- ☐ Yes
- ☐ No (please go to question 39)
- ☐ Don't know

© 2026 Ku NW et al. *JAMA Network Open*.

38. How often did you get to places in your neighbourhood, other than your own yard or apartment building?

- ☐ Less than once a week
- ☐ 1-3 times per week
- ☐ 4-6 times per week
- ☐ Daily
- ☐ Don't know

39. During the four weeks prior to your surgery, have you been to places outside your neighbourhood, but within your town?

- ☐ Yes
- ☐ No (please go to question 41)
- ☐ Don't know

40. How often did you get to places outside your neighbourhood, but within your town?

- ☐ Less than once a week
- ☐ 1-3 times per week
- ☐ 4-6 times per week
- ☐ Daily
- ☐ Don't know

41. During the four weeks prior to your surgery, had you been to places outside your town?

- ☐ Yes
- ☐ No (please go to question 43)
- ☐ Don't know

42. How often did you get to places outside your town?

- ☐ Less than once a week
- ☐ 1-3 times per week
- ☐ 4-6 times per week
- ☐ Daily
- ☐ Don't know

© 2026 Ku NW et al. *JAMA Network Open*.

43. Did you use aids or equipment, or need help from another person to get to other rooms of your home besides the room where you sleep?

- ☐ Yes personal assistance
- ☐ Yes equipment only
- ☐ No
- ☐ Did not leave my room
- ☐ Don't know

44. Did you use aids or equipment, or need help from another person to get to an area outside your home such as your porch, deck or patio, hallway (of an apartment building) or garage, in your own yard or driveway?

- ☐ Yes personal assistance
- ☐ Yes equipment only
- ☐ No
- ☐ Did not leave my home
- ☐ Don't know

45. Did you use aids or equipment, or need help from another person to get to places in your neighbourhood, other than your own yard or apartment building?

- ☐ Yes personal assistance
- ☐ Yes equipment only
- ☐ No
- ☐ Did not leave my home
- ☐ Don't know

46. Did you use aids or equipment, or need help from another person to get to places outside your neighbourhood, but within your town?

- ☐ Yes personal assistance
- ☐ Yes equipment only
- ☐ No
- ☐ Did not leave my neighbourhood
- ☐ Don't know

47. Did you use aids or equipment, or need help from another person to get to places outside your town?

- ☐ Yes personal assistance
- ☐ Yes equipment only
- ☐ No
- ☐ Did not leave my town
- ☐ Don't know

© 2026 Ku NW et al. *JAMA Network Open*.

48. Who helped you in the past month with (check all that apply)	**Laundry**	**Shopping**	**Cleaning**	**Preparing hot meals**	**Handling bills and keeping track of medications**
**Do not need help with this activity**	☐	☐	☐	☐	☐
**Spouse**	☐	☐	☐	☐	☐
**Daughter**	☐	☐	☐	☐	☐
**Son**	☐	☐	☐	☐	☐
**Daughter-in –law**	☐	☐	☐	☐	☐
**Son-in-law**	☐	☐	☐	☐	☐
**Stepdaughter**	☐	☐	☐	☐	☐
**Stepson**	☐	☐	☐	☐	☐
**Sister**	☐	☐	☐	☐	☐
**Brother**	☐	☐	☐	☐	☐
**Granddaughter**	☐	☐	☐	☐	☐
**Grandson**	☐	☐	☐	☐	☐
**Niece**	☐	☐	☐	☐	☐
**Nephew**	☐	☐	☐	☐	☐
**Cousin**	☐	☐	☐	☐	☐
**Neighbor**	☐	☐	☐	☐	☐
**Friend**	☐	☐	☐	☐	☐
**Ex-wife**	☐	☐	☐	☐	☐
**Ex-husband**	☐	☐	☐	☐	☐
**Other relative:** ______________	☐	☐	☐	☐	☐
**Other nonrelative:** ______________	☐	☐	☐	☐	☐

49 In the past month, <u>did you go without</u> at least once (see below) because it was too difficult to do by yourself and no one was available to help you or do that for you

Activity	**Yes**	**No**
**Clean laundry**	☐	☐
**Groceries or personal items**	☐	☐
**Hot meal**	☐	☐
**Paying a bill or banking**	☐	☐
**Made a mistake taking medicine**	☐	☐

© 2026 Ku NW et al. *JAMA Network Open*.

50. Who helped you in the past month with (check all that apply)

	Eating	Showering/ bathing/ washing up	Dressing	Using the toilet
I don't need help with this activity	☐	☐	☐	☐
Spouse	☐	☐	☐	☐
Daughter	☐	☐	☐	☐
Son	☐	☐	☐	☐
Daughter-in –law	☐	☐	☐	☐
Son-in-law	☐	☐	☐	☐
Stepdaughter	☐	☐	☐	☐
Stepson	☐	☐	☐	☐
Sister	☐	☐	☐	☐
Brother	☐	☐	☐	☐
Granddaughter	☐	☐	☐	☐
Grandson	☐	☐	☐	☐
Niece	☐	☐	☐	☐
Nephew	☐	☐	☐	☐
Cousin	☐	☐	☐	☐
Neighbor	☐	☐	☐	☐
Friend	☐	☐	☐	☐
Ex-wife	☐	☐	☐	☐
Ex-husband	☐	☐	☐	☐
Other relative: _________	☐	☐	☐	☐
Other nonrelative: ___________	☐	☐	☐	☐

51. In the past month, did you go without at least once (see below) because it was too difficult to do by yourself and no one was available to help you or do that for you

	Yes	No
Go without eating	☐	☐
Go without bathing/showering/washing up	☐	☐
Go without getting dressed	☐	☐
Accidently wet or soiled your clothes?	☐	☐

© 2026 Ku NW et al. *JAMA Network Open*.

52. Do you also receive help from someone you pay? By paid help, we mean paid by someone as part of a job.

- ☐ Yes
- ☐ No

b. If yes: what person(s)/service(s) do you pay to help you?_ ___________

c. If yes: how many hours a week do you receive help from this person/service?_ ______________

d. If yes: how much do you or your family pay per month for this person/services?_ _________________

© 2026 Ku NW et al. *JAMA Network Open*.

eMethods: Details on the sample sizes of the frail sub-study

The main FIT After Surgery study planned to include 2000 participants to ensure a representative sample. We estimated 45% of the total sample would score >3 CFS based on the work of team member Dr. McIsaac[3] and initial data from the main study.

Based on literature[16,20,45], the LSM decreases from baseline before surgery to approximately two months post-surgery and then partially recovers. Using previous studies, from baseline to two months there is a decline of 22 units and from two months to six months there is an increase of 10 units[16,20]. As our population is older and living with frailty, to ensure that there is sufficient power to detect a minimally clinical important difference (MCID) of five units in the two month- to six-month interval.[47] The standard deviation of the paired differences varies from 22.5 to 31.5 in this population[16]. Therefore, a sample of 329 patients was needed to achieve 80% power to detect a mean of paired differences of 5.0 with an estimated conservative standard deviation of differences of 31.5 and a significance level of 0.05. However, due to delays due to COVID-19 to start this Frail sub-study and the recruitment progress of the main FIT study which was over halfway before we could start our recruitment, we expanded our inclusion criteria to CFS 3 and higher instead of 4 and higher to have a larger potential sample size to recruit from. All main FIT study participants meeting our eligibility criteria were asked if they were interested in hearing more about our stub-study. If they indicated yes, our research coordinator contacted the older adult and explained the study and invited them to participate.

© 2026 Ku NW et al. *JAMA Network Open*.

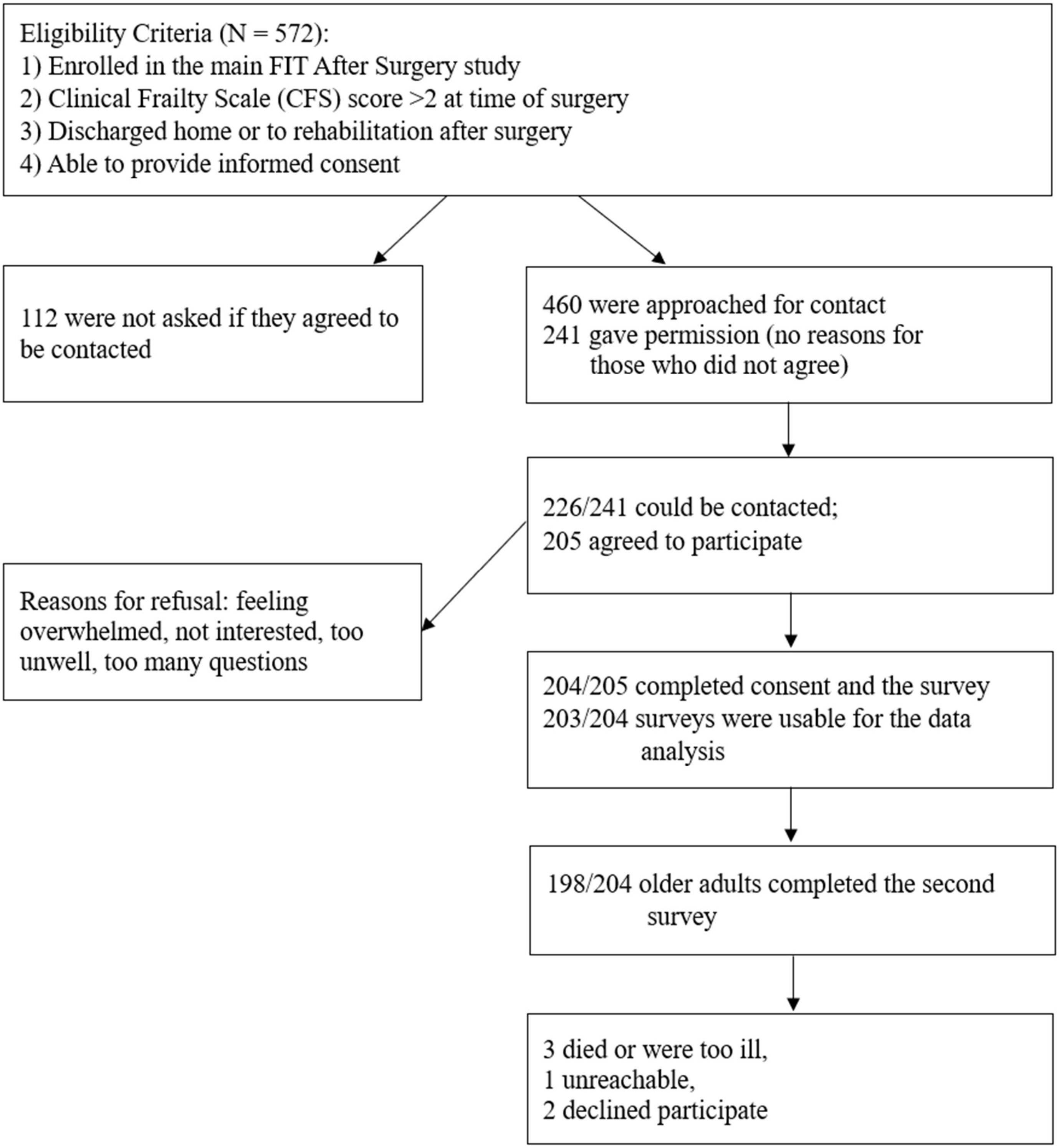

eFigure 1. Flow chart overview of recruitment and retention of older adults (n=205)

]

© 2026 Ku NW et al. *JAMA Network Open*.

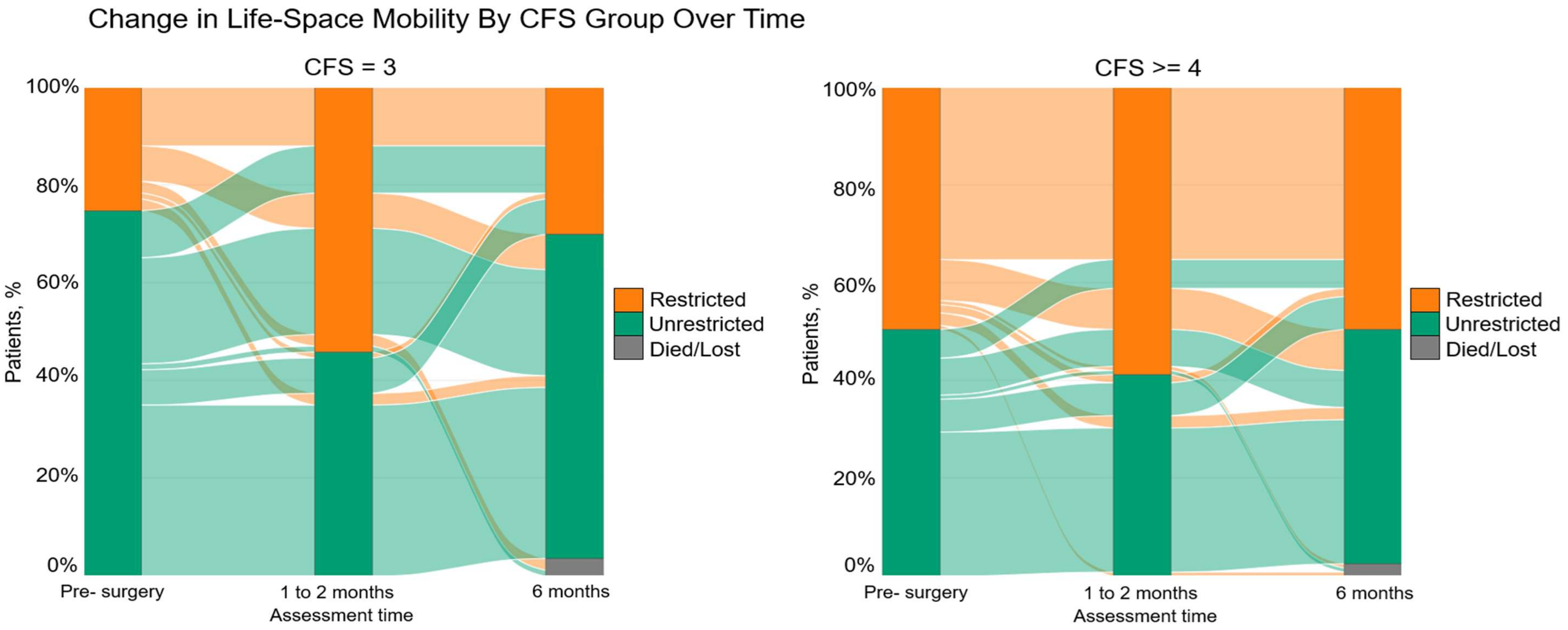

eFigure 2. Life-space mobility (LSM) status by clinical frailty scale (CFS) group from presurgery to 2 and 6 months after surgery.

© 2026 Ku NW et al. *JAMA Network Open*.

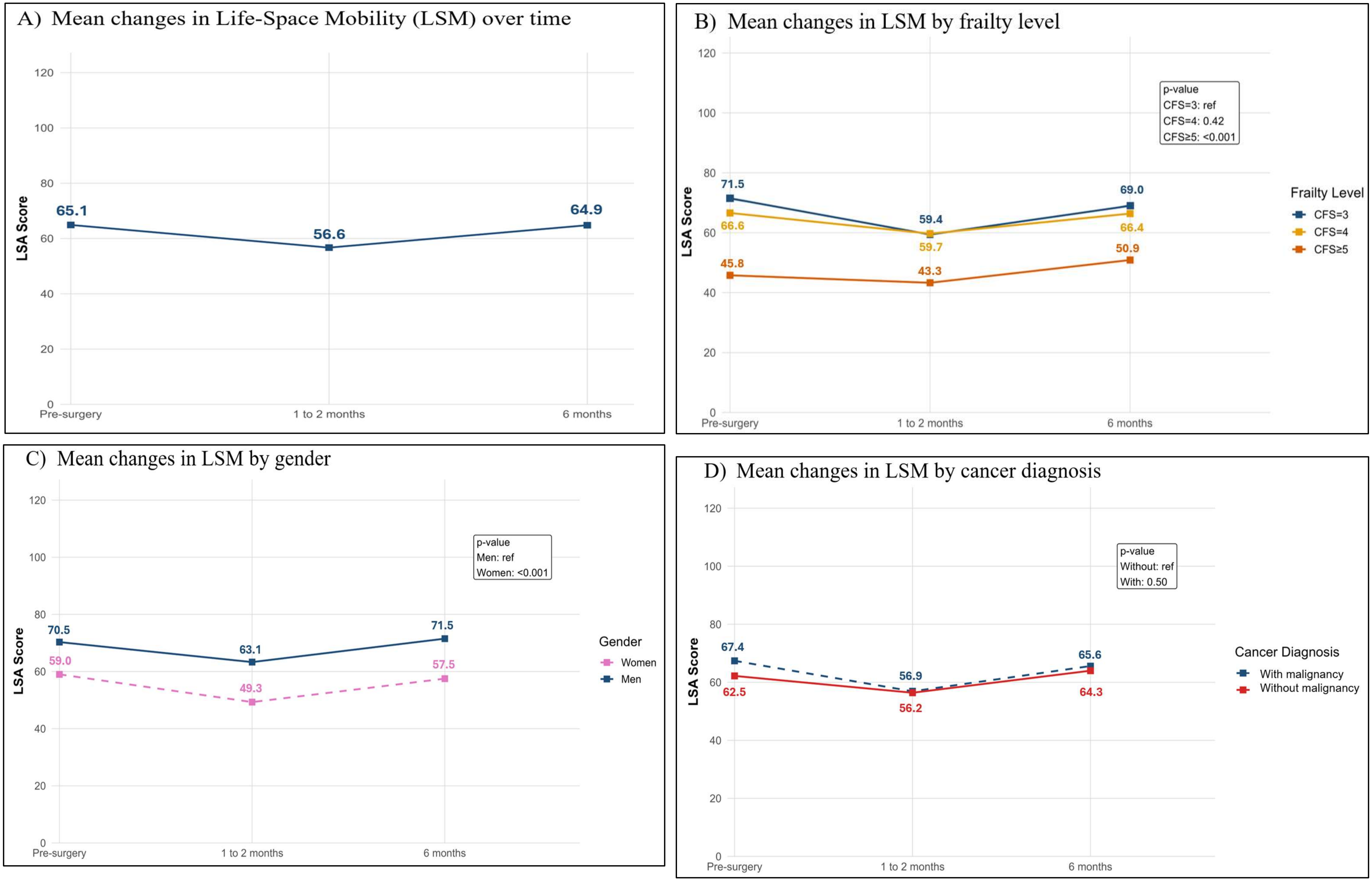

eFigure 3. Mean changes in Life Space Mobility (LSM) over time and stratified by frailty, gender, and cancer diagnosis.
CFS: Clinical Frailty Scale.

© 2026 Ku NW et al. *JAMA Network Open*.

eTable 1. Linear mixed-effect model for Life-Space Mobility (LSM) within 6 months now including education and living at home alone versus not (sensitivity analysis)

	Level	Estimated Coefficient (95% CI)	p-value
Gender	Men	ref	ref
	Women	-12.19 (-17.92, -6.47)	<0.001
Age (per 5-y increase)		-1.25 (-3.83, 1.34)	0.35
Clinical Frailty Scale	CFS 3	ref	ref
	CFS 4	-1.09 (-7.20, 5.01)	0.73
	CFS ≥ 5	-14.75 (-22.67, -6.83)	<0.001
Elevated Risk Surgery	No	ref	ref
	Yes	5.29 (-0.49, 11.08)	0.07
Time	Pre-surgery	ref	ref
	1 to 2 months	0.71 (-3.74, 5.16)	0.75
	6 months	6.15 (2.24, 10.06)	0.002
The need for support from formal or informal caregivers	No	ref	ref
	Yes	-12.10 (-16.34, -7.86)	<0.001
Education	At least some College/University	Ref	Ref
	Did not complete high school	-5.54 (-15.83, 4.75)	0.29
	Completed high school	-7.22 (-15.19, 0.75)	0.08
Living at home alone	No	Ref	Ref
	Yes	-2.32 (-8.91, 4.26)	0.49

© 2026 Ku NW et al. *JAMA Network Open*.

eTable 2. Multivariable mixed-effects logistic regression for restricted mobility (LSM < 60) within 6 months now including education and living at home alone vs not (sensitivity analyses)

	Level	Odds Ratio (95% CI)	p-value
Gender	Men	ref	ref
	Women	5.18 (2.45, 10.97)	<0.001
Age (per 5-y increase)		1.18 (0.86, 1.62)	0.32
Clinical Frailty Scale	CFS 3	ref	ref
	CFS 4	1.35 (0.65, 2.83)	0.42
	CFS ≥ 5	8.70 (3.04, 24.88)	<0.001
Elevated Risk Surgery	No	Ref	ref
	Yes	0.52 (0.26, 1.06)	0.07
Time	Pre-surgery	Ref	ref
	1 to 2 months	0.97 (0.46, 2.07)	0.94
	6 months	0.49 (0.24, 0.97)	0.05
The need for support from formal or informal caregivers	No	Ref	ref
	Yes	5.41 (2.70, 10.84)	<0.001
Education	At least some College/University	Ref	Ref
	Did not complete High School	3.05 (0.84, 11.05)	0.09
	Completed High School	3.90 (1.40, 10.83)	0.009
Living at home alone	No	Ref	Ref
	Yes	1.42 (0.64, 3.12)	0.39

© 2026 Ku NW et al. *JAMA Network Open*.

eTable 3. Multivariable logistic regression model for hospital readmission within 6 months (n=38) now including education and living at home alone versus not (sensitivity analyses)

	Level	Odds Ratio (95% CI)	p-value
Unmet needs	No	ref	ref
	Yes	1.96 (0.80, 4.75)	0.14
Life Space Mobility	Pre-surgery	1.01 (0.99, 1.02)	0.34
Clinical Frailty Scale	CFS 3	ref	ref
	CFS 4	0.84 (0.36, 1.96)	0.69
	CFS ≥ 5	0.91 (0.26, 2.89)	0.87
Elevated Risk Surgery	No	Ref	ref
	Yes	3.19 (1.43, 7.46)	0.006
Education	At least some College/University	Ref	Ref
	Did not complete High School	0.21 (0.01, 1.18)	0.15
	Completed High School	0.92 (0.30, 2.54)	0.88
Living at home alone	No	Ref	Ref
	Yes	0.72 (0.31, 1.69)	0.43

© 2026 Ku NW et al. *JAMA Network Open*.
