# Supplementary material for: Life-Space Mobility Trajectories After Elective Surgery in Older Adults
Source: JAMA Netw Open. 2026 Jul 22;9(7):e2624553. doi: 10.1001/jamanetworkopen.2026.24553 (PMC13392812; doi:10.1001/jamanetworkopen.2026.24553)
Supplement: Supplement 2. — Data Sharing Statement [file jamanetwopen-e2624553-s002.pdf]

Data Sharing Statement

Ku. Life Space Mobility Trajectories After Elective Surgery in Older Adults. *JAMA Netw Open*. Published July 22, 2026. doi:10.1001/jamanetworkopen.2026.24553

Data
Data available: No

Additional Information
Explanation for why data not available: We did not include this in our consent form for older adults and their caregivers.
